# The clinical course and outcomes of SARS-CoV-2 virus infection in children: a 24-week follow-up study in Sulaimaniyah, Iraq

**DOI:** 10.1186/s12887-023-04111-0

**Published:** 2023-06-17

**Authors:** Rozhan Nabaz Mohammed Sedik

**Affiliations:** 1 Department of Medical Education, College of Medicine, University of Sulaimani, Sulaimaniyah, Iraq; 2 Dr Jamal Ahmed Rashid Pediatric Teaching Hospital, Sulaimaniyah, Iraq

**Keywords:** COVID-19 pandemic, Persistent symptoms, Cohort study, Children infection

## Abstract

Most children infected with the SARS-CoV-2 virus have asymptomatic or mild disease with a short clinical course and excellent outcome; meanwhile, some children experienced persisting symptoms lasting > 12 weeks from the COVID-19 infection diagnosis. This study aimed to define the acute clinical course of SARS-CoV-2 virus infection and outcomes in children after recovery. This prospective cohort study was conducted on 105 children (aged < 16 years) with confirmed COVID-19 infection at Jamal Ahmed Rashid Teaching Hospital, Sulaimaniyah, Iraq, from July to September 2021. The symptomatic and suspicious cases of COVID-19 infection in children were confirmed by real-time reverse transcriptase-polymerase chain reaction (RT-PCR) on nasopharyngeal swabs. About 85.6% of children fully recovered at ≤ 4 weeks from initial COVID-19 infection diagnosis, 42% were hospitalized, while 15.2% reported long COVID-19 infection symptoms. The most commonly reported symptoms were fatigue (7.1%), hair fall (4.0%), lack of concentration (3.0%), and abdominal pain (2.0%). Children aged 11–16 showed a greater risk of long-term COVID-19 infection symptoms. We also observed a higher risk of long COVID infection symptoms in those who reported ongoing symptoms at 4–6 weeks of follow-up assessment (*p* = 0.01). Despite mild disease and complete recovery in most children, many suffered from long COVID infection symptoms.

## Introduction

Most children who suffer from coronavirus disease 2019 (COVID-19) infection have asymptomatic or mild illness with complete recovery [1]. However, severe pneumonia and systemic inflammatory response, known as Multisystem Inflammatory Syndrome in Children (MIS-C), occurred sporadically [2, 3]. In addition, some children have experienced persistent symptoms of the disease, which is "long COVID", also called post-COVID syndrome [4].

Long COVID infection is characterized by multisystem involvement. Many symptoms have been related to this condition, including fatigue, breathlessness, cough, anxiety, depression, palpitation, chest pain, myalgia, cognitive dysfunction (brain fog), and loss of smell [5]. Long COVID infection has been increasingly studied in adults, and significant mental and physical health impairment has been documented [6]. The incidence in adults was considerably higher than in children and adolescents, and it is estimated that 20–30% of adults had ongoing symptoms for weeks to months after acute SARS-CoV-2 virus infection [7].

Understanding of long COVID infection in children is evolving, and its frequency and severity are uncertain. There was no agreement on the clinical case definition and duration of symptoms of this condition in children while this study was in process. Until recently, an online three-phase Delphi process was conducted using a Lime survey followed by a virtual consensus meeting. They developed the following definition of long COVID infection in children and young people that could be used for research purposes: “*post-COVID-19 condition occurs in young people with a history of confirmed SARS-CoV-2 virus infection, with at least one persisting physical symptom for a minimum duration of 12 weeks after initial testing that an alternative diagnosis cannot explain. The symptoms impact everyday functioning may continue or develop after COVID-19 infection and may fluctuate or relapse over time”* [8].

Since the onset of the pandemic, the healthcare system in Sulaimaniyah has been strained, and knowledge of the burden of long COVID infection was scarce. Moreover, long COVID infection follow-up care for patients with lingering COVID-19 infection symptoms was not established because of the evidence gap. Hence, this study was conducted to determine the initial clinical course and, most notably, the sequelae after acute phase recovery to bring more attention to this condition.

## Patients and methods

### Sample size, study and design

**Fig. 1:**
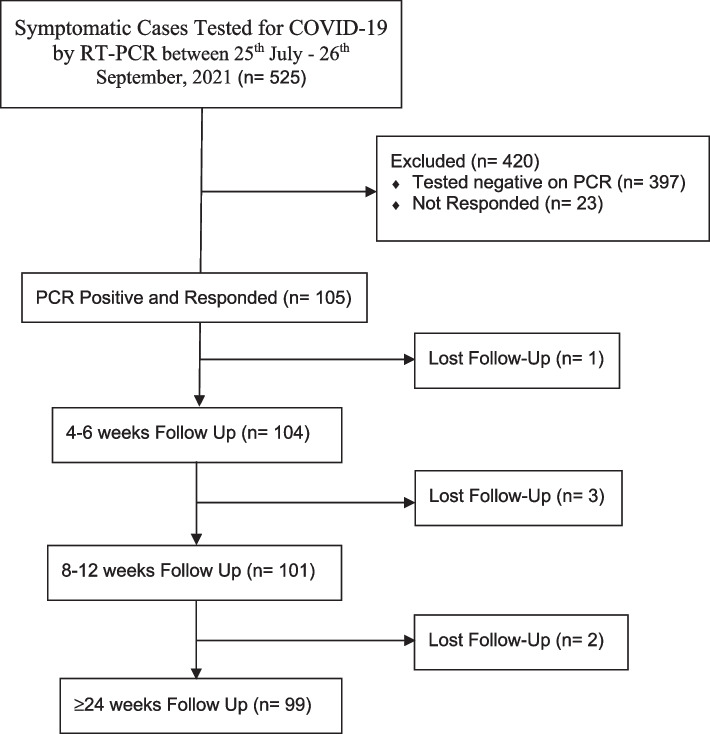
The participant’s flow diagram

This prospective cohort study was conducted on 105 children aged < 16 years who visited Jamal Ahmed Rashid Pediatric Teaching Hospital, Sulaimaniyah, Iraq, from July 25 to September 26 2021, corresponding with the period of a rapid surge of COVID-19 infection cases in the area (Fig. 1).

On nasopharyngeal swabs, the symptomatic and suspicious children with COVID-19 infection were confirmed by real-time reverse transcriptase-polymerase chain reaction (RT-PCR). Then, the caregivers were contacted by telephone and interviewed by a paediatrician with 10-year experience. The medical record of hospitalized patients was reviewed to confirm the caregiver's report on outcome and treatment. A special questionnaire was designed to inquire about children's socio-demographics and characteristics of their acute illness and for follow-up assessments. For this purpose, ISARIC COVID-19 Health and Wellbeing Initial Follow-Up Survey for Children was used with minor modifications to adapt to local requirements [9].

In the initial interview, data were collected thoroughly on children's socio-demographic characteristics, COVID-19 infection transmission risk factors, number of households/symptomatic household contact, comorbidities, acute stage of the disease (symptoms, duration of fever, treatment, and clinical outcome), and severity (hospitalization, pediatric intensive care (ICU) admission, oxygenation (PICU), continuous positive airway pressure (CPAP). Then, the acute severity of COVID-19 infection was determined based on the WHO classification [10].

### Inclusion criteria

Both hospitalized and nonhospitalized children with confirmed COVID-19 infection aged < 16 years, regardless of gender, ethnicity, or nationality, were enrolled in this study.

### Exclusion criteria

Children whose symptoms started > 30 days before the interview were excluded. Also, nonresponding parents were excluded from the follow-up assessment after three trials of telephone calls.

### Case definition

Cases whose symptoms resolved within four weeks were considered acute COVID-19 infection, those who remained symptomatic for 4–12 weeks were regarded as ongoing symptomatic, and those who reported at least one symptom beyond 12 weeks after initial testing were considered as long COVID infection [8, 11].

### Follow up assessment

The studied cases were followed up until the resolution of acute symptoms, and those with ongoing symptoms were contacted more than once until they recovered acute phase. After that, a follow-up telephone scheduled according to the time from COVID-19 infection initial testing (4–6 weeks, 8–12 weeks, and ≥ 24 weeks), and caregivers asked if their child had new or persistent symptoms within the last seven days (which was not present before COVID-19 infection). Additionally, caregivers reported the duration of symptoms, the degree of distress experienced by the children (not at all, only a little, quite a lot, a great deal), and any health problem after COVID-19 infection (multisystem inflammatory syndrome, Kawasaki disease, respiratory failure, asthma, myocarditis, diabetes, depression, anxiety, kidney problem, and other conditions).

### Statistical analysis

The collected data were entered into the Statistical Package for the Social Sciences (SPSS, version 23.0, IBM Corporation, Armonk, NY, USA). The Chi-square test was used for the correlation between variables. Descriptive statistics were presented as medians plus quartiles (interquartile range [IQR] 25^th^–75^th^ percentile) for continuous variables that did not have a normal distribution and as frequency/percentages for categorical variables. A *P*-value of < 0.05 was set as significant.

## Results

### Study population

A total of 525 children tested for COVID-19 infection using RT-PCR between July 25 to September 26 2021; however, only 128 cases tested positive, and 23 of them were excluded because of the wrong contact information or refusal to participate. Overall, this study enrolled 105 patients with a median age of 6.3 years and IQR of 4–12. Most of the children were males (51.4%), aged 0–5 years (42.9%), Kurdish nationality (96.2%), their contact with confirmed PCR-positive households (50.5%) was the leading risk factor and had a mild form of infection (91.4%). In comparison, comorbidities were reported in only 13.3%. At the same time, most of them (58.09%) were non-hospitalized (*n* = 61). However, significant differences (*p* < 0.05) were found between hospitalized and nonhospitalized patients for age, residency, comorbidities and severity of the COVID-19 infection (Table 1).

**Table 1:** Baseline characteristics of children with positive RT-PCR for SARS-CoV-2 infection

Socio-demographic characteristic	Total (Number, %)	Hospitalized (Number, %)	Non-Hospitalized (Number, %)	*P*-value
**Gender**				
Male	54 (51.4)	27 (61.4)	27 (44.3)	0.08
Female	51 (48.6)	17 (38.6)	34 (55.7)	
**Age group (Year)**				
0–5	45 (42.9)	30 (68.2)	15 (24.6)	< 0.001*
6–10	38 (36.2)	9.0 (20.5)	29 (47.5)	
11–16	22 (21.0)	5.0 (11.4)	17 (27.9)	
**Ethnicity**				
Kurd	101 (96.2)	44 (100.0)	57 (93.4)	0.08
Arab	4.0 (3.8)	0 (0.0)	4.0 (6.6)	
**Residency**				
Center of the city	87 (82.9)	32 (72.7)	55 (90.2)	0.03*
Outside the city	18 (17.1)	12 (27.3)	6.0 (9.8)	
**Risk factors**				
Unknown	20 (19.0)	10 (22.7)	10 (16.4)	0.3
Confirmed PCR-positive household contact	53 (50.5)	18 (41.0)	35 (57.4)	
Symptomatic household (family or relative)	26 (24.8)	14 (31.8)	12 (19.7)	
Childcare/school confirmed the contact	2.0 (1.9)	0.0 (0.0)	2.0 (3.3)	
Summer course	4.0 (3.8)	2.0 (4.5)	2.0 (3.3)	
**Comorbidities**	14 (13.3)	12 (27.3)	2.0 (3.3)	< 0.001*
Congenital cardiac disease	3.0 (2.9)			
Chronic respiratory disease (asthma)	1.0 (1.0)			
Neuromuscular disease	5.0 (4.8)			
Congenital obstructive renal disease	1.0 (1.0)			
Down syndrome	1.0 (1.0)			
Endocrine problem (primary hypoparathyroidism)	3.0 (2.9)			
**Severity of COVID-19**				
Mild	96 (91.4)	35 (79.5)	61 (100.0)	0.003*
Moderate	6.0 (5.7)	6.0 (13.6)	0.0 (0.0)	
Severe	2.0 (1.9)	2.0 (4.5)	0.0 (0.0)	
Critical	1.0 (1.0)	1.0 (2.3)	0.0 (0.0)	
**Total**	**105**	**44**	**61**	

^*^: Significant difference using the Chi-square test

### Clinical course and severity of acute COVID-19 infection

**Fig. 2:**
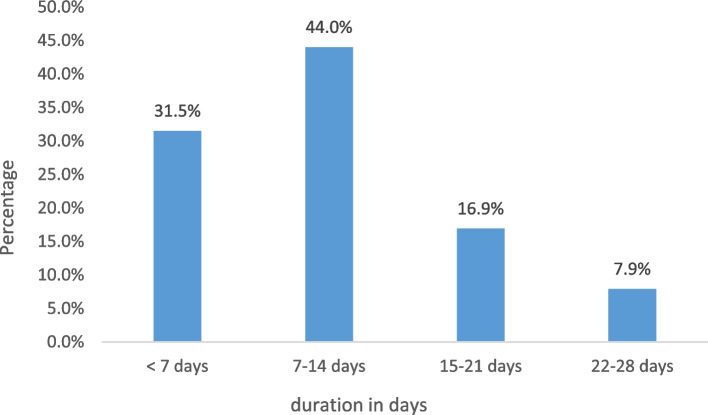
Duration of symptoms resolution in children during their acute COVID-19 infection

The most commonly reported symptoms were fever in most children (93.3%), fatigue (93.3%), poor appetite (92.4%), followed by respiratory symptoms (84.8%, especially cough), neurological symptoms (82.9%, especially headache), gastrointestinal symptoms (72.4%, especially diarrhoea), and musculoskeletal symptoms (47.6%, especially myalgia). In comparison, only 1.9% had cardiovascular symptoms (Table 2). Furthermore, in most infected children (44%), the acute symptoms were resolved within 7–14 days, while only 7.9% of their symptoms were resolved within 22–28 days (Fig. 2).

**Table 2:** Clinical symptoms during acute COVID-19 infection in children

Symptom	Frequency	%
**General**		
Fever	98	93.3
Rigor	12	11.4
Poor appetite	97	92.4
**Fatigue**	98	93.3
Mild fatigue	79	75.2
Cannot get out of bed	19	18.1
**Respiratory symptom**	89	84.8
Cough	62	59.0
Sore throat	35	33.3
Nasal congestion	45	42.9
Rhinorrhea	40	38.1
Hoarseness	7.0	6.7
Sneezing	34	32.4
Shortness of breath	15	14.3
Chest pain/tightness	8.0	7.6
**Cardiovascular symptoms**	2.0	1.9
Palpitation	2.0	1.9
**Gastrointestinal symptoms**	76	72.4
Abdominal pain	45	42.9
Nausea	31	29.5
Vomiting	42	40.0
Diarrhea	48	45.7
Decrease bowel motion	3.0	2.9
**Musculoskeletal symptoms**	50	47.6
Myalgia	50	47.6
Joint pain	3.0	2.9
**Neurological symptoms**	87	82.9
Headache	52	49.5
Dizziness on standing	19	18.1
Confusion/drowsiness	20	19.0
Seizure	2.0	1.9
Low mood/depressed/hopeless	13	12.4
Excessive crying	6.0	5.7
Lack of sleep	37	35.2
More sleep than usual	9.0	8.6
Tingling feeling/Pins and needles	2.0	1.9
Loss of taste	6.0	5.7
Altered taste/bitter	7.0	6.7
Loss of smell	11	10.5
**Other problem**		
Ear pain	8.0	7.6
Eye sore or pain	11	10.5
Red-eye (conjunctivitis)	18	17.1
Mouth ulcer	6.0	5.7
Skin Rash	10	9.5
Hair loss	3.0	2.9
**Total**	**105**	**100**

Moreover, the mean duration of children hospitalized was 3.0 ± 3.43 days, and most (63.6%) were hospitalized for only 1–2 days in the regular ward (97.7%). The most complicated symptoms in hospitalized children were pneumonia (11.4%) and seizure (4.5%). Regarding the treatment modes of the hospitalized children, 84.1% received antibiotics, 11.4% required oxygen therapy, and only one (2.3%) underwent CPAP treatment (7 months female), and none of them required mechanical ventilation (Table 3).

**Table 3:** Hospitalization course and treatment of children with COVID-19 infection

Characteristic of Hospitalization	Frequency	%
**Complication**		
Pneumonia	5.0	11.4
Seizure	2.0	4.5
**Length of hospital stay (Day)**		
1–2	28	63.6
3–5	10	22.7
≥ 6	6.0	13.6
Regular ward	43	97.7
Intensive care unit (ICU)	1.0	2.3
**Treatment**		
Antibiotics	37	84.1
Steroids	12	27.3
Antiviral (Remdesivir)	1.0	2.3
Oxygen therapy	5.0	11.4
CPAP	1.0	2.3
Mechanical ventilation	0.0	0.0
**Total**	**44**	**100**

### Follow-up assessment for persistent symptoms

After 4–6 weeks of acute COVID-19 infection diagnosis, only 15 out of 104 (14.4%) cases had ongoing symptoms (mostly females aged 11–16 years with comorbidities). The most common persistent symptoms were fatigue (6.7%), cough (2.9%), poor intake (2.9%), abdominal pain (2.9%), myalgia (2.0%), and headache (2.0%). Less than half of parents (46.7%) felt the symptoms caused their children quite a lot of distress. Whereas 8–12 weeks after a diagnosis of acute COVID-19 infection, only 20.8% of infected children (mostly females aged 11–16 with comorbidities) had persisting symptoms, and 15.8% reported only one symptom. The most common symptoms were hair fall (5.9%), fatigue (5.0%), myalgia (4.0%), poor intake (3.0%), lack of concentration (3.0%), and headache (2.0%). More than two third of parents (71.4%) felt the symptoms caused only a little distress to their child (Table 4).

**Table 4:** Follow-up assessment for persistent symptoms in children with COVID-19 infection

Variable	Duration after the COVID-19 diagnosis (Number, %)		
	**4–6 weeks**	**8–12 weeks**	** ≥ 24 weeks**
**Baseline cases**	104	101	99
**Full recovery**	89 (85.6)	80 (79.2)	84 (84.8)
**Persistent symptoms**	15 (14.4)	21 (20.8)	15 (15.2)
**One symptom**	7/104 (6.7)	16/101 (15.8)	11/99 (11.1)
**Two-Four symptoms**	8/104 (7.7)	5/101 (5.0)	4/99 (4.0)
Fatigue	7/104 (6.7)	5/101 (5.0)	7/99 (7.1)
Cough	3/104 (2.9)	0.0 (0.0)	0.0 (0.0)
Fever	1/104 (1.0)	0.0 (0.0)	0.0 (0.0)
Myalgia	2/104 (2.0)	4/101 (4.0)	1/99 (1.0)
Abdominal pain	3/104 (2.9)	1/101 (1.0)	2/99 (2.0)
Nausea	1/104 (1.0)	0.0 (0.0)	0.0 (0.0)
Vomiting	1/104 (1.0)	0.0 (0.0)	0.0 (0.0)
Diarrhea	1/104 (1.0)	1/101 (1.0)	0.0 (0.0)
Poor intake	3/104 (2.9)	3/101 (3.0)	1/99 (1.0)
Headache	2/104 (2.0)	2/101 (2.0)	1/99 (1.0)
Lack of concentration	0.0 (0.0)	3/101 (3.0)	3/99 (3.0)
Hypersomnia	0.0 (0.0)	1/101 (1.0)	1/99 (1.0)
Hair fall	1/104 (1.0)	6/101 (5.9)	4/99 (4.0)
Skin peeling	1/104 (1.0)	0.0 (0.0)	0.0 (0.0)
Sweating	0.0 (0.0)	1/101 (1.0)	1/99 (1.0)
Disturbed taste	0.0 (0.0)	1/101 (1.0)	1/99 (1.0)
Hand numbness and pain	0.0 (0.0)	1/101 (1.0)	0.0 (0.0)
**Does the symptom distress your child?**			
Not at all	0/15 (0.0)	0/21 (0.0)	0/15 (0)
Only a little	6/15 (40)	15/21 (71.4)	10/15 (66.7)
Quite a lot	7/15 (46.7)	4/21 (19.0)	2/15 (13.3)
A great deal	2/15 (13.3)	2/21 (9.5)	3/15 (20.0)
**Gender**			
Male	6/53 (11.3)	7/50 (14)	7/50 (14.0)
Female	9/51 (17.6)	14/51 (27.5)	8/49 (16.3)
***P*-value**	0.36	0.17	0.75
**Age (Year)**			
0-5	7/44 (15.9)	6/43 (14.0)	3/42 (7.1)
6-10	3/38 (7.9)	6/36 (16.7)	5/35 (14.3)
11-16	5/22 (22.7)	9/22 (40.9)	7/22 (31.8)
***P*-value**	0.27	0.03*	0.03*
**Severity of COVID-19**			
Mild	11/95 (11.6)	19/93 (20.4)	13/91 (14.3)
Moderate	1/6 (16.7)	1/5 (20.0)	1/5 (20.0)
Severe	2/2 (100.0)	1/2 (50.0)	1/2 (50.0)
Critical	1/1 (100.0)	0/1 (0.0)	0/1 (0.0)
***P*-value**	< 0.001*	0.73	0.53
**Hospitalization**			
Yes	8/43 (18.6)	8/42 (19.0)	5/41 (12.2)
No	7/61 (11.5)	13/59 (22.0)	10/58 (17.2)
***P*-value**	0.31	0.72	0.49
**Comorbidities**			
Yes	5/14 (35.7)	6/13 (46.2)	4/13 (30.8)
No	10/90 (11.1)	15/88 (17.0)	11/86 (12.8)
***P*-value**	0.01*	0.01*	0.09

^*^: Significant difference using the Chi-square test

Moreover, ≥ 24 weeks after a diagnosis of acute COVID-19 infection, 15.2% had ongoing symptoms (mostly females aged 11–16 years with comorbidities), of which 11.1% had only one symptom. The most common persistent symptoms were fatigue (7.1%), hair fall (4.0%), lack of concentration (3.0%), and abdominal pain (2.0%). However, most parents (66.7%) felt the symptoms caused little distress to their children. Overall, 89 (85.6%) cases were fully recovered at ≤ 4 weeks, and all the studied patients survived, with no recorded death (Table 4).

No significant difference was found between males and females in the prevalence of persisting symptoms. The adolescent group (11–16 years) reported more persisting symptoms at 8–12 weeks and 24-week follow-up assessment (*p* = 0.03). However, three cases with severe and critical illness had ongoing symptoms on initial follow-up (*p* < 0.001) but were insignificant in subsequent follow-ups. The rate of persisting symptoms did not vary significantly between hospitalized and nonhospitalized children (Table 4).

Children with pre-existing diseases had a higher probability of persisting symptoms (at 4–6 weeks and 8–12 weeks, *p* = 0.01) than children without. In addition, previously healthy participants were at lower risk for severe illness and hospitalization (*p* ≤ 0.001) (Table 5).

**Table 5:** Comparison between children with and without comorbidity

Characteristics		Comorbidity Number %	No Comorbidity Number %	*P*-value
**Severity of illness**	Mild	8/14 (57.1)	88/91 (96.7)	< 0.001*
	Moderate	4/14 (28.6)	2/91 (2.2)	
	Severe	2/14 (14.3)	0/91 (0.0)	
	Critical	0/14 (0.0)	1/91 (1.1)	
**Persistent Symptoms**	4–6 weeks	5/14 (35.7)	10/90 (11.1)	0.01*
	8–12 weeks	6/13 (46.2)	15/88 (17.0)	0.01*
	≥ 24 weeks	4/13 (30.8)	11/86 (12.8)	0.09
**Hospitalization**		12/14 (85.7)	32/91 (35.2)	< 0.001*
**Length of hospital stay (Day)**	1–2	5/12 (41.7)	23/32 (71.9)	0.16
	3–5	4/12 (33.3)	6/32 (18.8)	
	≥ 6	3/12 (25.0)	3/32 (9.4)	
**Treatment**	Antibiotics	11/12 (91.7)	26/32 (81.3)	0.40
	Steroids	5/12 (41.7)	7/32 (21.9)	0.13
	Antiviral	1/12 (8.3)	0/32 (0.0)	0.09
	Oxygen	3/12 (25.0)	2/32 (6.3)	0.08
	CPAP	0/12 (0.0)	1/32 (3.1)	0.53
**Intensive care unit (ICU) admission**		0/12 (0.0)	1/32 (3.1)	0.53

^*^: Significant difference using the Chi-square test

**Fig. 3:**
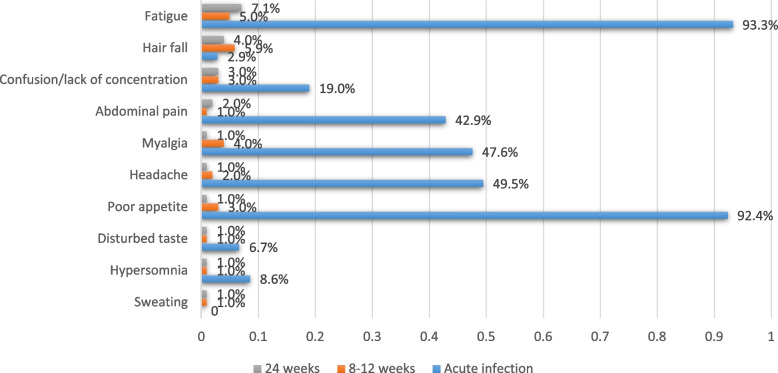
Frequency of long COVID symptoms in 24 weeks follow-up after initial diagnosis

Consequently, there was a significant reduction in the proportion of common persisting symptoms during acute COVID-19 infection and the last two follow-up assessments (Fig. 3), with a significant correlation between the presence of ongoing symptoms at 4–6 weeks’ assessment and persistent symptoms at both 8–12 weeks and 24-weeks follow-up assessment (*p* = 0.004 and 0.01, respectively) (Table 6).

**Table 6:** Comparison between ongoing symptoms after 4 weeks and persisting symptoms at 8–12 and 24 weeks’ assessment

Follow-Up Assessment	Ongoing symptoms 4–6 weeks		Total	*P*-value
	**Yes**	**No**		
**8–12 weeks’ persistent symptoms**				0.004*
Yes	7	14	21	
No	7	73	80	
			101	
** ≥ 24 weeks’ persistent symptoms**				0.01*
Yes	5	10	15	
No	8	76	84	
			99	

^*^: Significant difference using the Chi-square test

## Discussion

This study demonstrates that most children with COVID-19 infection, including hospitalized patients, had a mild clinical course with a low incidence of adverse outcomes; however, some participants suffered from multi-organ effects of persisting symptoms regardless of the severity of acute infection or hospitalization.

The current study observed that the most common acute clinical symptoms were fever/fatigue (93.3%), followed by poor appetite (92.4%), cough (59.0%), then headaches (49.5%), and myalgia (47.6%). These outcomes were also found in China [12]. The frequency of commonly reported symptoms was high because the participants were all symptomatic, and hospitalized patients were included. In addition, a considerable part of the cases reported diarrhoea, similar to other studies in China [2, 13].

On the other hand, most of the infected children recovered from acute symptoms within 4 weeks, and the largest proportion recovered in 14 days. The duration of hospitalization was short, and most were admitted for one to two days. Most of the admitted patients did not have complications; however, more than two-thirds of cases received antibiotics; this may reflect high fever in young children until the exclusion of severe bacterial illness, fever with symptoms of gastroenteritis (vomiting/diarrhoea) that initially treated until the COVID-19 infection test result revealed. These findings agreed with a study in Iran [14] and Saudi Arabia [15]. Moreover, this study was conducted while the Delta variant was the dominant circulation variant in the area, as described in a survey of molecular characterization of the SARS-CoV-2 virus strains in Iraq [16], and several studies showed that children were at higher risk of prolonged fever, hospitalization, and elevated inflammatory markers as compared to pre-Delta phase [17, 18].

The literature shows variability in the prevalence of persistent symptoms in children (1.6–70%) [19, 20]. In the present study, the rate of persistent symptoms declined from 20.8% at 8–12 weeks to 15.2% at 24 weeks of follow-up. Overall, 11.1% suffered from at least one symptom, and 4% reported two or more symptoms for more than 12 weeks and continued to ≥ 24 weeks from the initial COVID-19 infection diagnosis. In this regard, Roge et al. 2021 in Latvia conducted a study on 236 infected children, showing that long COVID symptoms were more common after COVID-19 infection (44.5%) [21]. An extensive cross-sectional survey in Italy reported persistent symptoms to affect < 20% of children [22], while another meta-analysis, which included 21 studies, showed that 25.24% of children and adolescents had symptoms of long COVID infection [23]. Moreover, a recent cohort study of 1884 SARS-CoV-2 virus-positive children with 90-day follow-up showed persistent symptoms in 9.8% and 4.8% of hospitalized and nonhospitalized children, respectively [5]. On the contrary, in England, a study reported that only 1.8% of children had symptoms at ≥ 8 weeks [1]. The variability in the rate of persistent symptoms is probably related to the use of different definitions, methodologies, and study designs. Still, all the studies support the presence of long COVID infection symptoms in children and highlight the importance of follow-up assessment after acute COVID-19 infection.

This study reported 10 long COVID infection symptoms, and fatigue was the most prevalent symptom, which agreed with that conducted in Turkey on 1007 children, which reported fatigue as the most common persistent symptom [24]. Another extensive systematic review reported fatigue as the most apparent persistent symptom [19]. Concentration difficulties and fatigue were among the most commonly reported persistent symptoms in the study on long COVID infection in children [4]. Cognitive disorders affected school-age children in our study, similar to another study's findings [21]. Furthermore, our studied children experienced hair fall 1–2 months after acute infection. Hence, hair fall is not a novel finding, as telogen effluvium is reported after stress and high fever, and it is usually transient; thus, among our studied cases, only one patient was disturbed a lot by hair fall, and the rest experienced only a little distress. In this respect, long COVID infection hair loss has been reported in adults and children as one of the long COVID infection symptoms in many other studies [23–25].

Other observed symptoms in infected children, such as abdominal pain, poor appetite, myalgia, disturbed taste, hypersomnia, and sweating, were also reported in previous studies among the common persistent symptoms [23, 26, 27]. However, unlike other studies, respiratory symptoms were not reported by the patients with long COVID infection symptoms; this might be because of the high rate of mild illness, including patients requiring hospitalization.

Following other studies, there was no significant difference in the long COVID infection symptoms rate between males and females [28]. However, the age group 11–16 years experienced more persistent symptoms, and similar findings were reported in a recent national cohort study in Denmark [29]. In addition, another study at Bashlyaeva Children’s Municipal Clinical Hospital in Russia found that older children were at higher risk for long COVID [9]. On the contrary, young children (0–5 years) had the least reported persistent symptoms that might be partly explained by the inability to express themselves and report symptoms like fatigue.

Previous studies identified hospitalization and severity of acute infection as risk factors for prolonged COVID-19 infection [4, 9, 23]. However, in this study, despite no significant difference in the rate of persisting symptoms regarding disease severity or hospitalization, we observed that participants with lingering symptoms for ≥ 4 weeks were more likely than those who recovered within four weeks to report persistent symptoms at 8–12 weeks and 24 weeks follow-up. Therefore, we think the duration of acute COVID-19 infection can predict later disease sequelae.

Regarding the disease severity, the proportion of moderate and severe or critical cases was low in this study which might affect the result's significance. A recent adult study in Spain showed a similar frequency of long COVID-19 infection symptoms among hospitalized and nonhospitalized patients 2 years after acute infection [30].

In the present study, at 4–6 and 8–12-weeks follow-up, children with comorbidities were at higher risk; however, this risk was not significant at 24 weeks of assessment. Similarly, in Italy, the pre-existing disease did not affect the prevalence of long COVID-19 infection [25]. In contrast, a cohort study in England and Wales showed a higher risk for long COVID-19 infection in children with pre-existing conditions. In line with several studies, children with comorbidities were more likely to experience severe illness and require hospitalization [31–33].

Finally, we observed that two participants with long COVID infection symptoms had neuromuscular disease and reported fatigue as a persisting symptom. Still, it is difficult to confirm without evaluation because the chronic illness renders them vulnerable to fatigue.

## Conclusions

Children can have severe COVID-19 infection requiring hospitalization or experience long COVID infection symptoms, but the rate remains relatively low. Most participants with persistent symptoms complained only a little, and cases that needed medical evaluation were mainly older children with neurological symptoms and fatigue. Moreover, this study highlights the importance of raising awareness among healthcare professionals and families about monitoring patients after acute COVID-19 infection until future studies identify pathogenesis and predictors of the long COVID infection.

### Limitations of the study

The main limitations were a small sample size, and a control group of children without COVID-19 infection was not included, which can more precisely estimate the prevalence and risk factors associated with long COVID infection. Moreover, in this study, the cases were closely followed through phone calls due to the pandemic lockdown and spread risk. Regarding hospitalized patients, their medical records were reviewed for confirmation. Still, for nonhospitalized, we relied solely on parents' and/or children's reports of the symptoms, and we did not examine or investigate to exclude other possibilities. Despite these limitations, this is the first study in Iraq to define the sequelae of COVID-19 infection and characteristics of the long COVID infection in different age groups and hospitalized and nonhospitalized children. We hope this study attracts the attention of healthcare professionals to long COVID infection and encourages follow-up assessments in primary health centres, especially for patients with severe and prolonged symptoms beyond four weeks.

## Data Availability

The data used to support the findings of this study are included in the article.
